# Selenium and silica nanostructure-based recovery of strawberry plants subjected to drought stress

**DOI:** 10.1038/s41598-020-74273-9

**Published:** 2020-10-19

**Authors:** Seyed Morteza Zahedi, Faezeh Moharrami, Saadat Sarikhani, Mohsen Padervand

**Affiliations:** 1 Department of Horticultural Science, Faculty of Agriculture, University of Maragheh, Maragheh, Iran; 2 Department of Horticulture, College of Aburaihan, University of Tehran, Tehran, Iran; 3 Department of Chemistry, Faculty of Science, University of Maragheh, Maragheh, Iran

**Keywords:** Plant sciences, Plant physiology, Plant stress responses

## Abstract

Drought is an important environmental stress that has negative effects on plant growth leading to a reduction in yield. In this study, the positive role of nanoparticles of SiO_2_, Se, and Se/SiO_2_ (SiO_2_-NPs, Se-NPs and Se/SiO_2_-NPs) has been investigated in modulating negative effects of drought on the growth and yield of strawberry plants. Spraying of solutions containing nanoparticles of SiO_2_, Se, and Se/SiO_2_ (50 and 100 mg L^−1^) improved the growth and yield parameters of strawberry plants grown under normal and drought stress conditions (30, 60, and 100%FC). Plants treated with Se/SiO_2_ (100 mg L^−1^) preserved more of their photosynthetic pigments compared with other treated plants and presented higher levels of key osmolytes such as carbohydrate and proline. This treatment also increased relative water content (RWC), membrane stability index (MSI) and water use efficiency (WUE). In addition, exogenous spraying of Se/SiO_2_ increased drought tolerance through increasing the activity of antioxidant enzymes including catalase (CAT), ascorbate peroxidase (APX), guaiacol peroxidase (GPX) and superoxide dismutase (SOD) as well as decreasing lipid peroxidation and hydrogen peroxide (H_2_O_2_) content. Increase in biochemical parameters of fruits such as anthocyanin, total phenolic compounds (TPC), vitamin C and antioxidant activity (DPPH) in strawberry plants treated with Se/SiO_2_ under drought stress revealed the positive effects of these nanoparticles in improving fruit quality and nutritional value. In general, our results supported the positive effect of the application of selenium and silicon nanoparticles, especially the absolute role of Se/SiO_2_ (100 mg L^−1^), on the management of harmful effects of soil drought stress not only in strawberry plants, but also in other agricultural crops.

## Introduction

Water shortage is a major environmental factor which limits the development and growth of plants and decreases the productivity and quality of crops^1,2^. Severe drought can also have an adverse effect on plant water use efficiency (WUE)^2^. The key physiological plant responses to drought stress are the synthesis and accumulation of compatible solutes, known as osmolytes or osmoprotectants which lower the cell water potential and enhance the water extraction capacity in environments with limited water resources^3^. Finding ways to improve drought tolerance is very useful for crop production, especially in sensitive species. The use of nanoparticles (NPs) has been shown to enhance the tolerance of plants to abiotic stress conditions much more quickly than approaches like genetic improvement^4^. Therefore, numerous studies have been conducted on several compounds to alleviate and improve the negative effects of environmental stresses on plants and enhance their quality^5–7^.

Strawberry (*Fragaria* × *ananassa* Duch.) is a small fruit with global significance because of its unique taste and the presence of a variety of biological compounds. In a chemical analysis of strawberry fruit, molecules such as antioxidants, phenolics, carotenoids, and ascorbic acid as well as minerals, vitamins, and sugars, are all found at high concentrations^8^. These compounds enhance fruit quality, and the antioxidant components present direct or indirect anti-allergic and antimicrobial activities as well as inhibiting activities of some physiological enzymes. In humans, compounds present in strawberry fruit help to reduce blood pressure and prevent stress^9^. Strawberry is among the most produced and consumed crops in Iran, such that its production has doubled during the last decade. Today, 0.6% (55,621 ton) of global strawberry production takes place in Iran^10^.

The reproductive and vegetative growth of strawberry is affected by environmental factors^11–13^. Drought is a major environmental stress which limits worldwide production of strawberry and affects its morphology as well as enzymatic and physiological activities^14^. Strawberry consumes a great amount of water because of its large leaf area, shallow root system and juicy texture. Consequently, it vegetative and reproductive growth are significantly damaged under drought stress, thereby reducing its biomass and crop yield^15^. The negative influences of osmotic stress are alleviated by ion exclusion, accumulation of soluble compounds such as proline and carbohydrates, and control of ion increase in roots through their transport to leaves^5^. Under oxidative stress, the antioxidant systems of plants, including specific enzymes catalase (CAT), ascorbate peroxidase (APX), peroxidase (POD) and superoxide dismutase (SOD) and non-enzymatic compounds (phenolics, carotenoids and ascorbic acid), are activated^16^.

A variety of approaches are applied relative to the improvement of plant tolerance to environmental stresses; the most widely reported of which is the application of exogenous compounds such as selenium (Se), silicon (Si), melatonin, etc.^5,6,17,18^. Among these compounds, Se and Si play important roles in the improvement of the tolerances of canola (*Brassica napus*), wheat (*Triticum aestivum*) and rice (*Oryza sativa*) to environmental stresses through increasing enzymatic antioxidant activities and maintaining photosynthetic apparatus performance^19–21^. Although Se and Si have not yet been identified as essential elements in the growth of plants, there have been reports on their favorable influences on plant growth and development, yield and abiotic stress tolerance^22^. Also, when Se and Si are applied at low concentrations, the growth parameters and quality values of pomegranate (*Punica granatum*) and orchid (*Phalaenopsis* and *Dendrobium*) were enhanced under controlled conditions, indicating that these elements are essential for both stress adaptation and growth and development of plants^23,24^. In addition, the application of novel Se/SiO_2_ NPs composition seems to have greater influences.

The depletion of water resources in agricultural areas, along with climate change, are the current challenges of agriculture and will remain so in the future. Because of the importance of strawberry in Iran and other countries, substances like Si and Se should be investigated for protective abilities and to alleviate the unfavorable influences of drought stress. The effects of synthesized recombinant nanoparticles, including both silica and selenium called Se/SiO_2_-NPs_,_ are being investigated relative to increased growth and development as well as modifying drought stress. Therefore, the main goal of this work was to achieve fundamental knowledge on the influences of foliar spray on strawberry with NPs containing Si and Se (SiO_2_-NPs, Se-NPs and especially Se/SiO_2_-NPs) under drought stress conditions. We further investigate the influences of NPs on the quality and yield of fruits, as well as the leaf concentration of chlorophyll fluorescence, photosynthetic pigments, anti-oxidative enzymes, oxidative markers and water use efficiency in plants grown under three different drought levels.

## Results

### Fresh and dry weight of root and shoot

**Figure 1:**
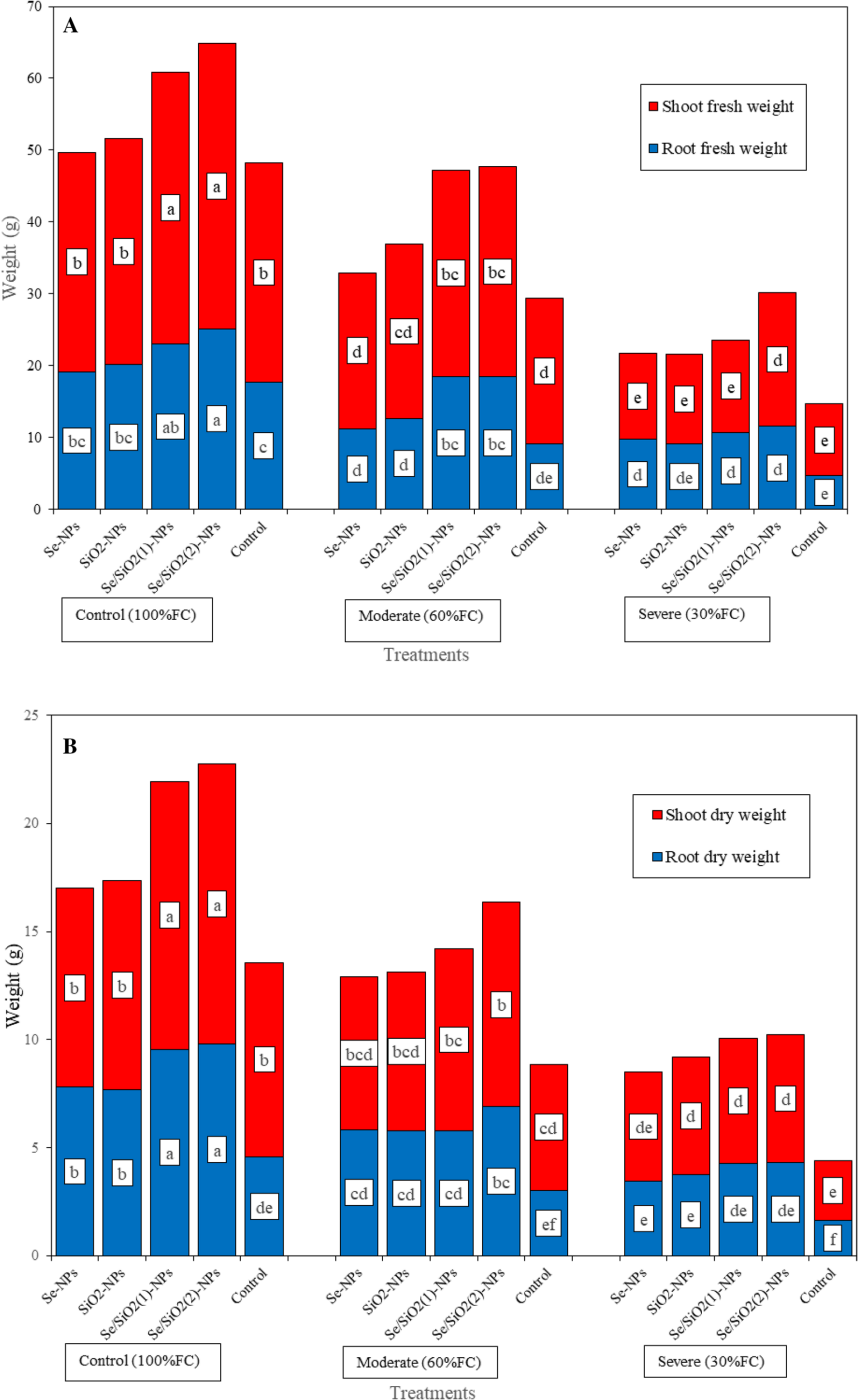
Effect of foliar application of nanoparticles containing selenium and silica on the growth parameters of strawberry plants under drought stress conditions. (**A**) Fresh weight of root and shoot; (**B**) Dry weight of root and shoot.

Fresh and dry weights of roots and shoots decreased with the increase of drought stress such that under severe drought stress, fresh and dry weights of roots as well of those of shoots decreased by 73/64/67/69%, respectively, compared with normal condition (Fig. 1A,B). At all drought stress levels, spraying with Se-, SiO_2_- and Se/SiO_2_-NPs increased the amounts of these parameters compared with water sprayed plants, with Se/SiO_2_-NPs having the stronger effect. Under non-stress conditions, spraying with Se/SiO_2_-NPs (100 mg L^−1^) increased fresh and dry weights of roots (42% and 113%, respectively) as well as those of shoots (29% and 43%, respectively) as compared to water sprayed plants. Under severe stress, spraying Se/SiO_2_-NPs (100 mg L^−1^) increased all four parameters by 144/167/87/112%, respectively.

### Leaf photosynthetic pigments and chlorophyll fluorescence

Amounts of chl a, chl b, total chlorophyll and carotenoid decreased with increasing drought stress (Table 1). Under severe drought stress the amounts of chl a, chl b, total chl and carotenoid decreased significantly (61/70/63/67%, respectively) compared with normal conditions. At all stress levels, spraying a solution containing Se-, SiO_2_- and Se/SiO_2_-NPs on strawberry plants increased the concentrations of photosynthetic pigments compared to plants sprayed with only water (Table 1). At all three stress levels (control, moderate, severe) Se/SiO_2_-NPs (100 mg L^−1^) was most effective at increasing the contents of chl a, chl b, total chl and carotenoid as compared with plants sprayed with only water.

**Table 1:** Effect of foliar application of nanoparticles containing selenium and silica on contents of photosynthetic pigments and chlorophyll fluorescence in strawberry plants under drought stress conditions.

Treatments		Chl *a *(mg g^−1^ FW)	Chl *b *(mg g^−1^ FW)	Total Chl (mg g^−1^ FW)	CARs (mg g^−1^ FW)
Drought (D)	NPs (mg L^−1^)				
Control (100%FC)	Control	9.85cd	3.69b	15.53cd	3.13c–f
	Se	11.22bc	4.20b	15.42bc	4.23a–c
	SiO_2_	11.11bc	4.16b	15.27bc	4.27a–c
	Se/SiO_2_(1)	11.93ab	4.43b	16.36b	4.44ab
	Se/SiO_2_(2)	13.16a	5.87a	19.04a	5.13a
Moderate (60%FC)	Control	6.95ef	2.11de	9.06e	2.16f
	Se	8.92d	3.62b	12.54d	3.20c–f
	SiO_2_	8.49de	3.31bc	11.80d	3.16c–f
	Se/SiO_2_(1)	8.96d	3.68b	12.65d	3.46b–e
	Se/SiO_2_(2)	9.40cd	4.19b	13.60cd	3.95b–d
Severe (30%FC)	Control	3.84g	1.10e	4.94f	1.01g
	Se	5.99f	1.83de	7.82e	2.77ef
	SiO_2_	5.44fg	1.79de	7.23e	2.67ef
	Se/SiO_2_(1)	5.36fg	2.00de	7.37e	2.93d–f
	Se/SiO_2_(2)	7.05ef	2.40cd	9.45e	3.45b–e
*Significance*					
*D*		**	**	**	**
*NPs*		*	*	*	*
D*NPs		*	*	*	*

Treatments		*F*_*0*_	*F*_*m*_	*F*_*v*_	*F*_*v*_*/F*_*m*_
Drought (D)	NPs (mg L^−1^)				
Control (100%FC)	Control	0.64d	3.37bc	2.72b	0.80ab
	Se	0.61d	3.33bc	2.72b	0.82ab
	SiO_2_	0.61d	3.29bc	2.71b	0.87ab
	Se/SiO_2_(1)	0.62d	3.82ab	2.72b	0.71ab
	Se/SiO_2_(2)	0.64d	4.11a	3.89a	0.95a
Moderate (60%FC)	Control	0.78d	2.32de	1.37cd	0.59bc
	Se	0.83cd	2.76cd	1.54cd	0.55b–d
	SiO_2_	0.84cd	2.78cd	1.52cd	0.57bc
	Se/SiO_2_(1)	0.86cd	2.89cd	1.63cd	0.57bc
	Se/SiO_2_(2)	0.96c	2.95cd	2.01bc	0.68ab
Severe (30%FC)	Control	1.06bc	1.07f	0.18e	0.17e
	Se	1.25b	1.68ef	0.57e	0.36c–e
	SiO_2_	1.27b	1.68ef	0.60e	0.36c–e
	Se/SiO_2_(1)	1.88a	2.44d	0.58e	0.26de
	Se/SiO_2_(2)	1.97a	2.63cd	0.92de	0.35c–e
*Significance*					
*D*		**	**	**	**
*NPs*		*	**	*	ns
D*NPs		*	*	*	*

Values represent the means ± standard errors of three independent replications (*n* = 3). Different letters within the same column indicate significant differences at *P* < 0.05 among the treatments, according to Duncan’s multiple range test. Se/SiO_2_(1: 50 mg L^−1^); Se/SiO_2_(2: 100 mg L^−1^); Chlorophyll a, Chl *a*; Chlorophyll b, Chl *b*; Total chlorophyll, Total Chl; Carotenoids, CARs; Fresh weight, FW; Minimum fluorescence, *F*_*0*_; Maximum fluorescence, *F*_*m*_; Variable fluorescence, *F*_*v*_; Maximum quantum efficiency of photosystem II, *F*_*v*_*/F*_*m*_*.*

Regarding chlorophyll fluorescence parameters, with an increase in drought stress, *F*_*0*_ increased but other photosynthetic parameters including *F*_*m*_, *F*_*v*_, and *F*_*v*_*/F*_*m*_ decreased (Table 1). Drought stress increased *F*_*0*_ (65%) and decreased *F*_*m*_, *F*_*v*_, *F*_*v*_*/F*_*m*_ (68/93/78%, respectively) compared to normal condition. Under moderate and severe drought stress, foliar application of Se-, SiO_2_- and Se/SiO_2_-NPs increased chlorophyll fluorescence parameters, with Se/SiO_2_-NPs having better effects (Table 1). At all stress levels, spraying with Se/SiO_2_-NPs (100 mg L^−1^) increased the amounts of *F*_*0*_, *F*_*m*_, *F*_*v*_, and *F*_*m*_*/F*_*v*_ by 0.36/23/85%, 22/26/144%, 43/46/394% and 18/15/102%, respectively, compared to plants sprayed with only water.

### Leaf total soluble carbohydrates, proline, membrane stability index and relative water content

The amounts of leaf carbohydrate and proline increased with increasing drought stress (Table 2). Under severe drought stress the amounts of carbohydrate and proline increased significantly (43 and 103%, respectively) compared with normal condition. At all drought stress levels, spraying of the plants with Se-, SiO_2_- and Se/SiO_2_-NPs increased the values of both parameters compared to spraying with water. Se/SiO_2_-NPs (100 mg L^−1^) had the most beneficial effects such that carbohydrate and proline increased in amounts by 91/84/79% and 20/62/49%, respectively, in sprayed plants.

**Table 2:** Effect of foliar application of nanoparticles containing selenium and silica on the osmolytes, relative water content, membrane stability index, oxidative markers and enzymatic antioxidants in strawberry plants under drought stress conditions.

Treatments		Carbs (mg g^−1^ FW)	Pro (mg g^−1^ FW)	MSI (%)	RWC (%)	MDA (nM g^−1^ FW)
Drought (D)	NPs (mg L^−1^)					
Control (100%FC)	Control	82.94g	0.23f	80.55ab	86.77a	1.19e
	Se	153.99c–e	0.24f	82.15a	86.66a	1.11e
	SiO_2_	108.96fg	0.24f	81.19ab	85.19a	1.10e
	Se/SiO_2_(1)	158.97c–e	0.26ef	83.29a	86.74a	0.95e
	Se/SiO_2_(2)	159.04c–e	0.28ef	84.29a	87.66a	1.17e
Moderate (60%FC)	Control	97.47fg	0.34d–f	53.19de	48.41cd	2.40c
	Se	155.71c–e	0.40c–e	57.90cd	61.78bc	2.01cd
	SiO_2_	130.68d–f	0.39c–e	56.67cd	60.47bc	1.94cd
	Se/SiO_2_(1)	170.66b-d	0.55b	67.95bc	72.50ab	1.68de
	Se/SiO_2_(2)	180.16a–c	0.56b	73.28ab	75.80ab	1.49de
Severe (30%FC)	Control	119.16e–g	0.47b–d	27.84g	28.74e	4.09a
	Se	179.06a–c	0.55b	38.66fg	44.61d	3.14b
	SiO_2_	172.43bc	0.52bc	34.03g	39.38de	3.16b
	Se/SiO_2_(1)	200.70ab	0.56b	40.36e–g	48.53cd	3.13b
	Se/SiO_2_(2)	213.76a	0.70a	50.98d–f	69.35b	2.01cd
*Significance*						
*D*		**	**	**	**	**
*NPs*		**	*	*	*	*
D*NPs		**	**	**	*	**

Treatments		H_2_O_2_ (nM g^−1^ FW)	CAT (Unit mg^−1^ prot)	APX (Unit mg^−1^ prot)	GPX (Unit mg^−1^ prot)	SOD (Unit mg^−1^ prot)
Drought (D)	NPs (mg L^−1^)					
Control (100%FC)	Control	13.76d	0.31d	1.01g	0.13f	0.30e
	Se	13.75d	0.32d	1.20g	0.14f	0.34de
	SiO_2_	13.73d	0.31d	1.13g	0.12f	0.35de
	Se/SiO_2_(1)	13.86d	0.33d	1.22g	0.14f	0.35de
	Se/SiO_2_(2)	13.91d	0.34d	1.26g	0.15f	0.37de
Moderate (60%FC)	Control	22.48bc	0.43cd	2.51f	0.32e	0.49c–e
	Se	17.53cd	0.59b–d	2.76ef	0.50d	0.47c–e
	SiO_2_	17.64cd	0.50b–d	2.75ef	0.51d	0.47c–e
	Se/SiO_2_(1)	16.16d	0.59b–d	3.50de	0.56cd	0.56cd
	Se/SiO_2_(2)	14.12d	0.83b	4.04cd	0.70b	0.81b
Severe (30%FC)	Control	42.87a	0.52b–d	4.13cd	0.49d	0.69bc
	Se	27.43b	0.69bc	4.96bc	0.63cd	0.81b
	SiO_2_	28.13b	0.69bc	4.42cd	0.60cd	0.89b
	Se/SiO_2_(1)	24.20b	0.72bc	5.51b	0.79b	0.84b
	Se/SiO_2_(2)	16.97cd	1.28a	8.19a	0.98a	1.11a
*Significance*						
*D*		**	**	**	**	**
*NPs*		*	*	*	*	*
D*NPs		*	*	**	**	*

Values represent the means ± standard errors of three independent replications (*n* = 3). Different letters within the same column indicate significant differences at *P* < 0.05 among the treatments, according to Duncan’s multiple range test. Se/SiO_2_(1: 50 mg L^−1^); Se/SiO_2_(2: 100 mg L^−1^); *Carbs* Total soluble carbohydrates, *Pro* Proline, *RWC* Relative water content, *MSI* Membrane stability index, *H*_*2*_*O*_*2*_ Hydrogen peroxide, *MDA* Malondialdehyde, *CAT* Catalase, *APX* Ascorbate peroxidase, *GPX* Guaiacol peroxidase.

The leaf MSI and RWC decreased with increasing drought stress (Table 2). Severe drought stress significantly decreased MSI and RWC (65 and 66%, respectively) compared with control plants. At all drought stress levels, spraying of plants with Se-, SiO_2_- and Se/SiO_2_-NPs increased the values of these two parameters compared to spraying with water, with Se/SiO_2_-NPs (100 mg L^−1^) sprays having a better effect. At all stress levels, comparing Se/SiO_2_-NPs sprayed and water sprayed plants revealed that spraying with Se/SiO_2_-NPs (100 mg L^−1^) increased MSI and RWC by 4/37/83% and 1/56/141%, respectively.

### Leaf malondialdehyde contents and hydrogen peroxide

The amounts of leaf MDA and H_2_O_2_ increased with drought stress compared with control plants (Table 2). Drought stress significantly increased MDA and H_2_O_2_ (242 and 211%, respectively). At all drought stress levels, spraying plants with Se-, SiO_2_- and Se/SiO_2_-NPs decreased the values of MDA and H_2_O_2_ compared to spraying with water. Se/SiO_2_-NPs (100 mg L^−1^) was most effective at effecting decreases in these parameters. Comparing Se/SiO_2_-NPs sprayed and water sprayed plants under moderate and severe stresses revealed that spraying with Se/SiO_2_-NPs (100 mg L^−1^) decreased MDA and H_2_O_2_ by 37/49% and 37/60%, respectively.

### Leaf catalase, ascorbate peroxidase, guaiacol peroxidase and superoxide dismutase

The activities of enzymes CAT, APX, GPX and SOD in leaves increased with drought stress compared to control plants. Severe drought increased the amounts of these parameters by 65/313/266/130%, respectively (Table 2). At all drought stress levels, spraying of plants with Se-, SiO_2_- and Se/SiO_2_-NPs increased the activities of these enzymes compared to spraying with water. Comparing Se/SiO_2_-NPs sprayed and water sprayed plants revealed that spraying with Se/SiO_2_-NPs (100 mg L^−1^) increased the activities of enzymes CAT, APX, GPX and SOD by 8/94/154%, 26/61/98%, 15/118/100% and 23/64/61%, respectively.

### Growth, firmness and fruit yield parameters

The number of leaves and leaf area decreased with increasing drought stress and significant decreases in these parameters were noted under the most severe drought stress treatment (Table 3; Supplementary File S1). At all drought stress levels, spraying of plants with Se-, SiO_2_- and Se/SiO_2_-NPs increased the number and area of leaves compared with plants sprayed with only water. As with previously reported parameters, Se/SiO_2_-NPs (100 mg L^−1^) had the most beneficial effect. At all treatment levels, plants treated with Se/SiO_2_-NPs (100 mg L^−1^) had higher numbers leaves and more leaf area compared with those sprayed only with water by 15/51/96% and 17/41/110%, respectively.

**Table 3:** Effect of foliar application of nanoparticles containing selenium and silica on the leaf and fruit yield parameters and firmness in strawberry plants under drought stress conditions.

Treatments		Number of leaves per plant	Leaf area (cm^2^)	Number of inflorescences per plant	Number of flowers per plant	Number of fruits per plant	Fruit size (g)	Fruit firmness (N)	Fruit weight (g)	Yield (g)
Drought (D)	NPs (mg L^−1^)									
Control (100%FC)	Control	8.43a–d	60.35a–c	1.66e–f	14.00c	7.33bc	13.63cd	5.14d	13.36a–c	90.55c–e
	Se	8.56a–c	62.69a–c	2.66bc	14.66bc	9.76b	13.70cd	5.16bc	13.93ab	132.54bc
	SiO_2_	8.46a–d	61.50a–c	2.66bc	14.33c	9.33b	13.33cd	5.14bc	12.91ab	119.62b–d
	Se/SiO_2_(1)	8.74ab	66.03ab	3.33b	18.33ab	10.00b	16.66bc	6.13b	15.18a	151.10b
	Se/SiO_2_(2)	9.73a	70.99a	4.33a	21.66a	15.00a	39.00a	7.60a	15.20a	231.63a
Moderate (60%FC)	Control	5.33e–g	43.84d–g	0.86f–i	8.16de	3.13d–f	9.40de	4.29c	8.86cd	27.26f–h
	Se	6.80b–f	52.00b–e	1.56d-g	11.66cd	4.93c–e	11.63cd	4.53c	11.81a–c	58.64e–g
	SiO_2_	6.73b–f	48.00c–f	1.59d–f	11.33cd	4.13de	11.43cd	4.50c	11.45bc	47.44e–h
	Se/SiO_2_(1)	7.83a–d	55.46b–d	1.90c–e	12.66c	5.30c–e	13.00cd	4.57c	12.02a–c	63.45e–g
	Se/SiO_2_(2)	8.06a–d	62.03a–c	2.33cd	14.70bc	5.93cd	20.30b	5.70b	13.12ab	79.31d–f
Severe (30%FC)	Control	3.03g	18.97i	0.10i	1.43f	0.60f	5.09e	2.15d	3.07e	0.64h
	Se	5.40e–g	29.81g–i	0.50hi	4.02f	2.60ef	8.50de	3.13d	6.33de	16.78gh
	SiO_2_	5.16fg	26.58hi	0.46hi	3.92f	2.50ef	8.20de	3.08d	6.00de	15.31gh
	Se/SiO_2_(1)	5.83d–f	33.98f–h	0.66g–i	4.09f	3.26d–f	10.96cd	3.18d	7.22d	23.61gh
	Se/SiO_2_(2)	5.96c–f	39.93e–h	1.33e–h	5.33ef	3.60de	13.66cd	3.24d	8.92cd	31.74f–h
*Significance*										
*D*		**	**	**	**	**	**	**	**	**
*NPs*		*	*	*	*	*	*	*	*	*
D*NPs		*	*	*	*	*	**	*	*	**

Values represent the means ± standard errors of three independent replications (*n* = 3). Different letters within the same column indicate significant differences at *P* < 0.05 among the treatments, according to Duncan’s multiple range test. Se/SiO_2_(1: 50 mg L^−1^); Se/SiO_2_(2: 100 mg L^−1^).

The numbers of inflorescences, flowers and fruit decreased with increases in drought stress (Table 3; Supplementary File S1). When compared with control plants, the amounts of decrease were 76/89/91%, respectively. At all drought stress levels, spraying of plants with Se-, SiO_2_- and Se/SiO_2_-NPs increased the amounts of all three parameters compared with plants sprayed with only water. Under the three conditions of normal and moderate and severe stress, comparing plants sprayed with Se/SiO_2_-NPs (100 mg L^−1^) and water revealed that the number of inflorescence, flower and fruit were increased by 160/169/233%, 54/80/272% and 104/89/500%, respectively.

Fruit size, fruit weight and yield also decreased with increasing levels of drought stress (Table 3). Under severe stress, the amount of decrease was 62/75/99% compared with normal condition. Under the three treatment conditions, spraying with Se/SiO_2_-NPs increased the amounts of all three parameters. Comparing control and Se/SiO_2_-NPs (100 mg L^−1^) treated plants revealed spraying increased fruit size, fruit weight, and yield 186/115/168%, 23/48/190% and 155/190/426%, respectively.

Fruit firmness decreased with the increase in drought stress compared to fruit from control plants (Table 3). Severe stress decreased fruit firmness by 58% compared with control plants. At all drought stress levels, spraying with a Se/SiO_2_-NPs solution increased fruit firmness compared with water sprayed plants. Se/SiO_2_-NPs (100 mg L^−1^) spraying resulted in 48/32/50% increase at all three stress levels.

### Fruit anthocyanin, total phenolic compounds, ascorbic acid content and antioxidant activity

The contents of anthocyanin and total phenolic compounds increased under severe stress compared to normal condition such that the values of these two parameters increased by 328 and 137%. Conversely, the contents of vitamin C and DPPH decreased under severe stress compared to normal condition such that the values of these parameters decreased by 62 and 48%, respectively (Table 4). Under control conditions, Se/SiO_2_-NPs (100 mg L^−1^) spraying increased the contents of anthocyanin, total phenol, vitamin C, and DPPH by 180/125/49/3%, respectively. Under severe drought condition, all four parameters were increased by 55/100/ 67/11% by Se/SiO_2_-NPs spraying compared to water spraying (Table 4).

**Table 4:** Effect of foliar application of nanoparticles containing selenium and silica on the anthocyanin, total phenolic compounds, ascorbic acid (Vit C) content and antioxidant activity in strawberry plants under drought stress condition.

Treatments		Anthocyanin (mg 100 g^-1^ FW)	TPC (mg GAE g^−1^ FW)	Vit C (mg 100 g^-1^ FW)	DPPH (%)
Drought (D)	NPs (mg L^−1^)				
Control (100%FC)	Control	5.36h	0.72f	60.00b–d	48.70d
	Se	10.40gh	1.31ef	72.33b	48.84d
	SiO_2_	10.26gh	1.26ef	70.33bc	45.36e
	Se/SiO_2_(1)	11.66gh	1.62e	73.25b	49.18cd
	Se/SiO_2_(2)	13.06fg	1.64e	89.66a	50.16bc
Moderate (60%FC)	Control	20.07ef	1.58e	46.66d–f	50.34b
	Se	33.33bc	1.86de	55.66b–d	51.08ab
	SiO_2_	33.00bc	1.80de	52.66c–e	51.10ab
	Se/SiO_2_(1)	35.56b	1.88de	62.57b–d	50.56b
	Se/SiO_2_(2)	44.00a	2.39cd	70.33bc	51.99a
Severe (30%FC)	Control	23.00de	1.72e	22.33g	25.68h
	Se	27.96b–d	2.85a–c	33.00fg	25.68h
	SiO_2_	27.63cd	2.78bc	32.08fg	25.28h
	Se/SiO_2_(1)	30.90bc	3.26ab	33.96fg	27.41g
	Se/SiO_2_(2)	35.66b	3.45a	37.33e–g	28.51f
*Significance*					
*D*		**	**	**	**
*NPs*		*	**	**	*
D*NPs		**	**	**	**

Values represent the means ± standard errors of three independent replications (*n* = 3). Different letters within the same column indicate significant differences at *P* < 0.05 among the treatments, according to Duncan’s multiple range test. Se/SiO_2_(1: 50 mg L^−1^); Se/SiO_2_(2: 100 mg L^−1^); Total phenolic compounds, TPC; Ascorbic acid, Vit C; antioxidant activity, DPPH.

### Water use efficiency

**Figure 2:**
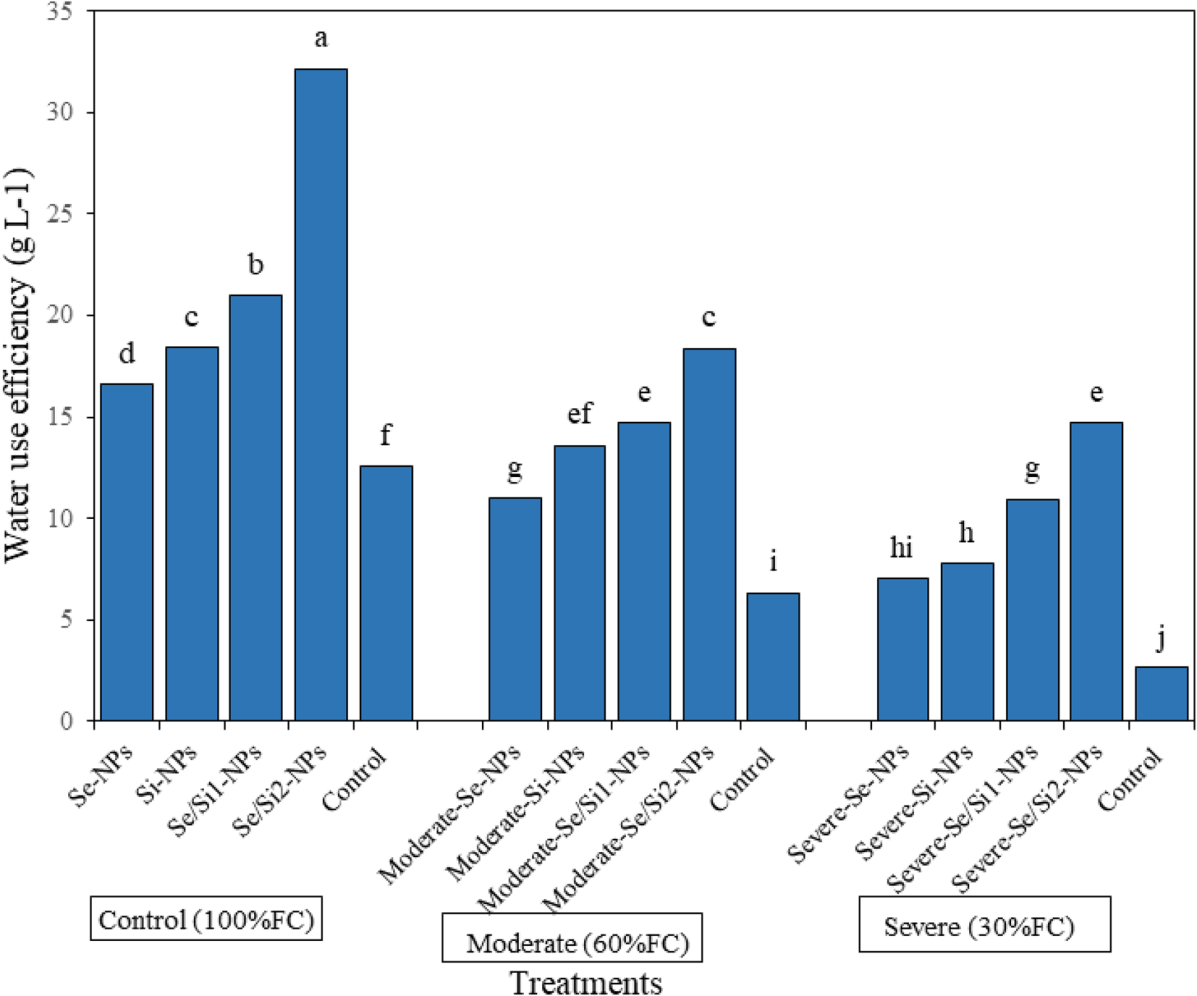
The influence of foliar sprays of selenium (Se), silicon dioxide (SiO_2_) and Se/SiO_2_ NPs on the water use efficiency (WUE = marketable yield per liter water used) of strawberry plants under different drought levels.

Leaf WUE decreased with drought stress compared with control plants. Under severe drought stress a 79% decrease in WUE was noted (Fig. 2). At all drought stress levels, spraying of plants with Se-, SiO_2_- and Se/SiO_2_-NPs increased the WUE compared to spraying with water. Comparing Se/SiO_2_-NPs sprayed and water sprayed plants under normal, moderate and severe stresses revealed that spraying with Se/SiO_2_-NPs (100 mg L^−1^) increased WUE by 155/190/452%, respectively.

### Multivariate analysis of NPs-treated strawberry plants under normal and drought stress conditions

**Figure 3:**
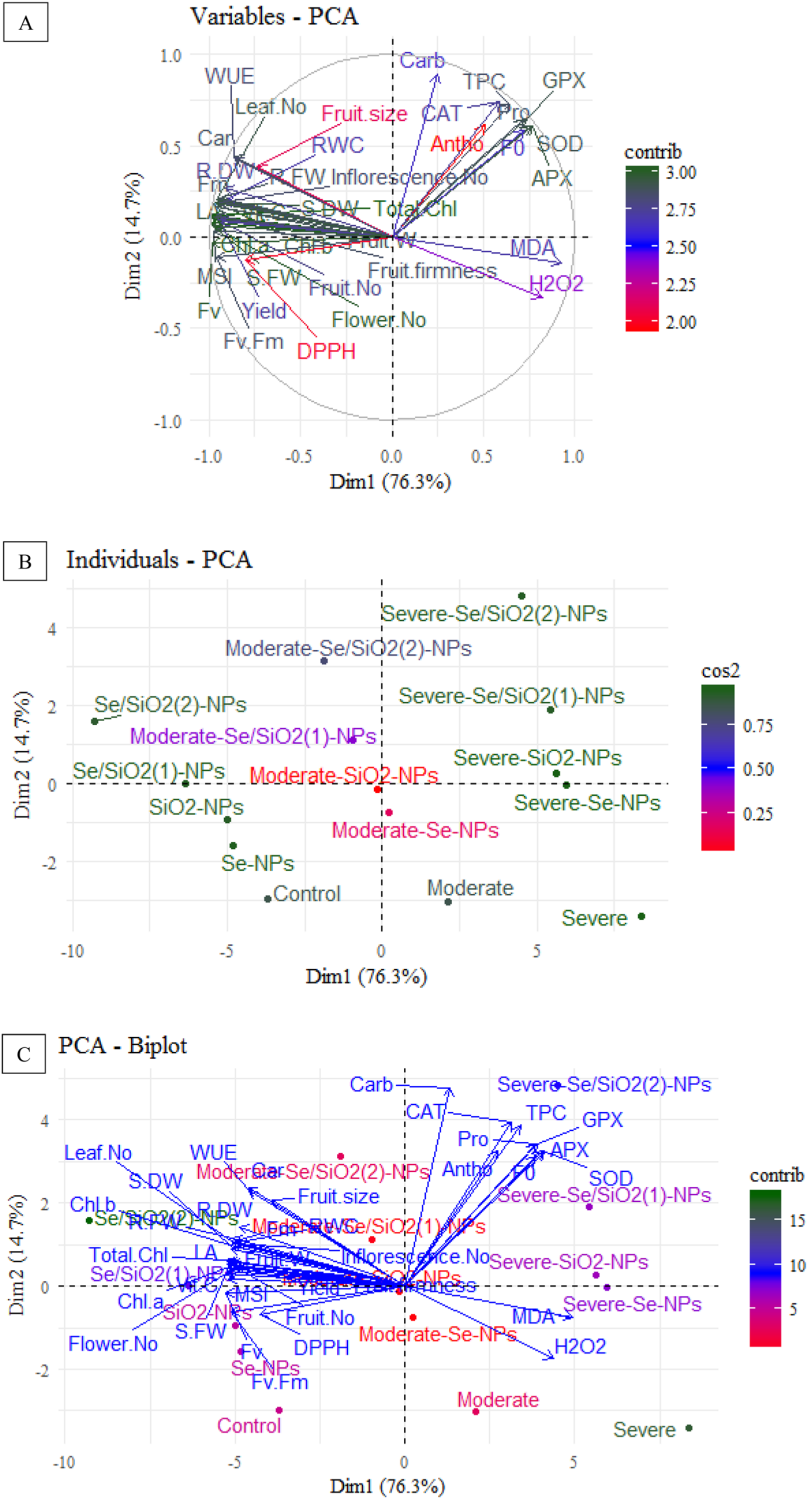
Principal component analysis (PCA) of treatments with selenium (Se), silicon dioxide (SiO_2_) and Se/SiO_2_ NPs and variable trait relationship in strawberry plants under normal and various drought stress conditions. (**A**) PCA loading plot of PC1 and PC2 of the evaluated variable traits, circles denote the most correlated variables. (**B**) PCA individual plot of selenium (Se), silicon dioxide (SiO_2_) and Se/SiO_2_ (1: 50; 2:100 mg L^−1^) NPs on strawberry plants under normal (control, 100%FC), moderate (60%FC) and severe (30%FC) drought conditions. (**C**) PCA biplot of treatment-variable associations where the lines originating from the center denote positive or negative correlations of different variables. The evaluated variables included Chlorophyll a, (Chl a); Chlorophyll b, (Chl b); Total chlorophyll, (Total Chl); Carotenoids, (CARs); Minimum fluorescence, *F*_*0*_; Maximum fluorescence, *F*_*m*_; Variable fluorescence, *F*_*v*_; Maximum quantum efficiency of photosystem II, *F*_*v*_*/F*_*m*_; *Carbs* Total soluble carbohydrates, *Pro* Proline, *RWC* Relative water content, *MSI* Membrane stability index, *H*_*2*_*O*_*2*_ Hydrogen peroxide, *MDA* Malondialdehyde, *CAT* Catalase, *APX* Ascorbate peroxidase, *GPX* Guaiacol peroxidase, *SOD* Superoxide dismutase, *TPC* Total phenolic compounds, *Vit C* Ascorbic acid, *DPPH* antioxidant activity, *WUE* Water use efficiency, *Dim1* dimension1, *Dim2* dimension2, *Contrib* contribution, *Cos2* squared cosine.

All 37 measured traits including morphological, physiological, biochemical and yield parameters were classified under two major principal component axes (PC1 and PC2) with 91% of total variance being accounted. Most of the investigated traits were classified as PC1 and therefore they had higher proportion of the variance (76.3%) while a lower proportion of variance (14.7%) was classified as PC2 (Fig. 3A). PCA plot classified samples into five major groups: (1) strawberries grown under normal condition; (2) strawberries grown under drought stress levels; (3) strawberries grown under normal condition with Se-, SiO_2_- and Se/SiO_2_-NPs spraying; (4) strawberries grown under moderate stress condition with Se-, SiO_2_- and Se/SiO_2_-NPs spraying; and (5) strawberries grown under moderate stress condition with Se-, SiO_2_- and Se/SiO_2_-NPs spraying (Fig. 3B). All morphological, WUE, photosynthetic pigments, and yield parameters had positive compliance with Se/SiO_2_-NPs while H_2_O_2_ and MDA accompanied strawberries grown under moderate and severe stress conditions (Fig. 3C). Also, carbohydrate, proline and oxidative enzymes accompanied Se/SiO_2_-NPs sprayed plants exposed to severe drought stress.

**Figure 4:**
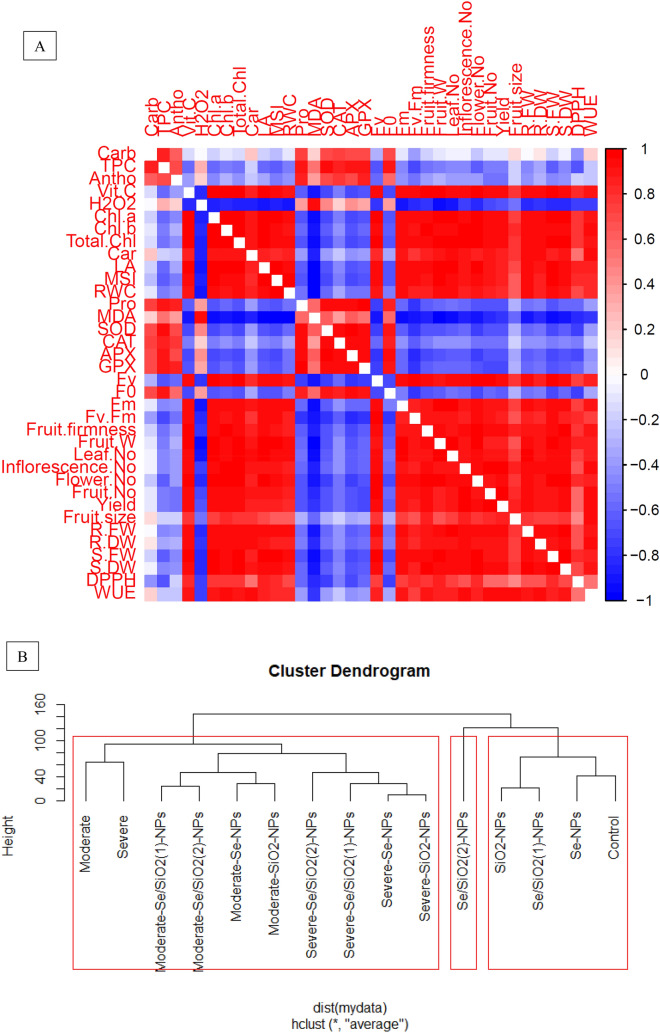
Pearson correlation analysis and dendrogram clustering of treatments with selenium (Se), silicon dioxide (SiO_2_) and Se/SiO_2_ NPs and variable trait relationship in strawberry plants grown under normal and different drought stress conditions. (**A**) Heatmap of Pearson correlation coefficient (r) values of variable traits, where the colored scale denotes positive (blue) or negative (red) correlations and the values ‘r’ coefficient were in the range of − 1.0 to 1.0. (**B**) Dendrogram clustering of selenium (Se), silicon dioxide (SiO_2_) and Se/SiO_2_ (1:50; 2:100 mg L^−1^) NPs in strawberry plants under normal (control, 100% FC), moderate (60% FC) and severe (30% FC) drought conditions. The evaluated variables included *Chl a* Chlorophyll a, *Chl b* Chlorophyll b, *Total Chl* Total chlorophyll, *CARs* Carotenoids, *F*_*0*_ Minimum fluorescence, *F*_*m*_ Maximum fluorescence, *F*_*v*_ Variable fluorescence, *F*_*v*_*/F*_*m*_ Maximum quantum efficiency of photosystem II, *Carbs* Total soluble carbohydrates, *Pro* Proline, *RWC* Relative water content, *MSI* Membrane stability index, *H*_*2*_*O*_*2*_ Hydrogen peroxide, *MDA* Malondialdehyde, *CAT* Catalase, *APX* Ascorbate peroxidase, *GPX* Guaiacol peroxidase, *SOD* Superoxide dismutase, *TPC* Total phenolic compounds, *Vit C* Ascorbic acid, *DPPH* antioxidant activity, *WUE* Water use efficiency.

Pearson correlation analysis showed a positive correlation between yield and yield parameters with photosynthetic pigments and morphological traits. Also, a positive correlation was observed between fruit biochemical and morphological traits as well as photosynthetic pigments. On the other hand, *F*_*0*_, H_2_O_2_, MDA and oxidative enzymes had a negative correction with morphological traits, photosynthetic pigments, yield parameters and yield. Water use efficiency was positively correlated with yield and yield parameters. It also had a direct and positive correlation with osmolytes, photosynthetic pigments and *Fv* (Fig. 4A).

Cluster analysis and dendrogram investigations showed two main classes for strawberry fruits sprayed with Se-, SiO_2_- and Se/SiO_2_-NPs under drought stress (Fig. 4B). Clade I contained strawberry plants grown under moderate and severe stress conditions and also plants grown under similar conditions sprayed with Se- and SiO_2_-NPs as well as Se/SiO_2_-NPs. Clade II contained control plant and those sprayed with Se-, SiO_2_- and Se/SiO_2_-NPs under normal conditions.

## Discussion

Drought is a harmful environmental^2^ issue and is one of the main factors responsible for decreases in growth and yield of agricultural crops around the world^25^. Drought stress decreases the yield and quality of fruits through changing the amounts and activities of photosynthetic pigments as well as osmolyte content and enzyme activity^2^. Recently, nanotechnology has found its way into agricultural systems. Application of minerals as nanoparticles in different shapes and sizes has high potential in increasing performance^4^ and modulating environmental stress^5^. Although the positive effects of SiO_2_-NPs and Se-NPs spraying on agricultural crops has been well established, the combinational effect of Se/SiO_2_-NPs has not yet been investigated.

### Fresh and dry weight of root and shoot

Morphological parameters, including fresh and dry weight of root and shoot, decreased under drought stress. Spraying of Se-, SiO_2_- and Se/SiO_2_-NPs alleviated the negative effects of this stress. Significant decreases of growth parameters under drought stress have been reported for different species such as lettuce^26^ and barley^27^. Decreases of growth parameters may be due to the decrease of RWC and consequent shrinkage of cells, decrease of meristematic cell division, leaf growth reduction, leaf production blockage, senescence acceleration and leaf drop^28^. Also, water stress can directly affect the biochemical processes involved in photosynthesis and indirectly decrease the entrance of carbon dioxide into stomata, which close when subjected to drought. Therefore, photosynthetic material transfer is affected by drought and photosynthesis is limited which decreases the vegetative growth of plants^29^. The positive effects of Si^30^ and Se^31^ have been reported in drought tolerance and fresh and dry weight of different species such as maize (*Zea mays*). Growth stimulation due to Si and Se under different environmental stress conditions has been assigned to increased photosynthesis and antioxidative enzyme activities^22^. Probably, in the presence of these elements, assimilate production is increased and the activity of antioxidant enzymes such as CAT and SOD are also increased resulting in the modulation of negative effects of stress and increase of vegetative growth^32^.

### Leaf photosynthetic pigments and chlorophyll fluorescence

The amount of photosynthetic pigments under drought stress conditions was significantly decreased compared to control plants, but spraying with Se-, SiO_2_- and especially Se/SiO_2_-NPs decreased these factors to a lower extent under drought stress. One reason for chlorophyll reduction during drought stress is that drought stress induces the production of active oxygen species which in turn destroys and decreases pigments. On the other hand, chlorophyll molecules decompose within chloroplasts and the thylakoid structure disappears^33,34^. Previous studies showed that Si and Se increase chlorophyll pigment content in different plants under stress and normal conditions^33,35^. It seems that these elements protect chloroplast structure against severe oxidative damage such as destruction of both grana and stroma lamellae, and increase the biosynthesis of photosynthetic pigments by protecting chloroplastic enzymes^33^. Probably, these elements act as cofactors in many enzymatic reactions involved in the biosynthetic pathways of the chloroplast^36,37^.

Fluorescence chlorophyll parameters, except *F*_*0*_, decreased significantly under drought stress; however, spraying with solutions containing Se-, SiO_2_- and especially Se/SiO_2_-NPs decreased them at a lower rate. To evaluate the effect of stress and estimate average quantum efficiency of photosystem II (QII), chlorophyll fluorescence parameters have been widely used. It seems that chlorophyll fluorescence indicates thylakoid membrane integrity and relative efficiency of electron transfer from photosystem II to photosystem I^38^. In fact, lower *F*_*0*_ values mean better photosynthetic activity^39^. Wright et al. (2009) reported the increase of *F*_*0*_ parameter under drought stress due to the loss of thylakoid membrane integrity^40^. In this research, the amount of *F*_*v*_ in normal and stress conditions decreased due to the inhibition of electrons, prevention of electron transfers from photosystem II to electron acceptance point in the Quinone (QA and QC) molecules and blockage of photo-oxidation of photosystem II^41^. Also, parameter *F*_*v*_*/F*_*m*_ is evaluated as an efficient tool in the discovery of damages to the photosynthetic system before it is revealed in the morphology of the plants. Decrease of *F*_*v*_*/F*_*m*_ in this experiment may be due to the damage of chloroplasts which is also confirmed by the reduction of chlorophyll content. The positive effects of Si^42^ and Se^43^ on fluorescence parameters under stress conditions have been reported for different plants. These elements increase the efficiency of light application by helping light transfer to active photosynthetic mesophyll^42^. Si protects photochemical reactions from harmful effects of stress through increasing *F*_*v*_*/F*_*m*_ ratio^44^.

### Leaf osmolyte status and WUE, MDA and H_2_O_2_ contents and antioxidant enzyme activities

Drought stress increased the amounts of carbohydrate and proline in strawberry leaves and spraying with solutions containing Se-, SiO_2_- and especially Se/SiO_2_-NPs, increased the levels of osmolytes. Increases of osmolytes in different plants such as maize^45^ and trifoliate orange rootstock^46^ under drought stress have been previously reported. Under drought conditions and due to a decrease of available water content, photosynthesis decreased, consequently decreasing the production of dry matter and assimilates^47^. Also, proline acted as a nitrogen and carbon source in plants under drought stress and the tolerance of plants against stress was increased. Under drought conditions, proline plays essential roles in protecting osmotic potential, scavenging of free radical and ROS, protecting molecules against denaturation, and adjusting cell pH. These events depend on plant species, stress duration, growth stage and stress intensity^47^. The positive effect of Se^48^ and Si^49^ in adjusting the amounts of osmolytes for modulating harmful effects of stress have been reported. It seems that these inorganic elements increase the production of soluble sugars through enhancing photosynthesis, which is probably effective as osmolytes in preserving water equilibrium, in addition to general growth stimulation.

MSI and RWC decreased significantly through the increase in drought stress, but spraying solutions containing Se-, SiO_2_- and especially Se/SiO_2_-NPs, increased their contents. Membranes are the first place in the cell which is influenced under stress conditions and the ability of plants to protect the integrity of membranes under drought stress determines the tolerance of plant to drought stress. Under drought stress, the water potential of soil decreases and plants prevent transpiration phenomenon using different mechanisms such as closing stomata, increasing stomatal resistance, and decreasing stomatal conductivity^50^. It was observed that Se/SiO_2_-NPs spraying increased both RWC and MSI in strawberry plants. It seemed that the consumption of Se and Si increased the amount of antioxidants and decreased free radical activities in plants which in turn increased cell membrane stability and improved water potential under normal and stress conditions^51^. Research has shown that Si plays a key role in protecting RWC under stress conditions^52^. Mateos-Naranhi et al. showed that the improving effect of Si on hydration status of plants could help decrease transpiration or phytolith deposition under epidermis cells of leaves and stems, which decreased waste of water from cuticle layers^53^. It seems, a well-ticked layer of silicon dioxide should help postpone water loss; thereby, it can increase WUE^54^.

While severe drought induced oxidative stress and increased the levels of H_2_O_2_ and MDA, spraying with Se-, SiO_2_- and especially Se/SiO_2_-NPs decreased the harmful effects of stress. Also, drought stress increased the activity of antioxidant enzymes including CAT, APX, GPX and SOD in the leaves of strawberry plants and spraying of the nanoparticles increased the activity of these enzymes. Research has shown that severe drought induces oxidative stress due to the accumulation of ROS, which in turn induces free radicals that could not be controlled in damaging cell components and, eventually leading to cell death^55^. Increase of MDA, H_2_O_2_ and other antioxidant enzymes have been reported for different plant species under drought stress^55^. Some elements such as Si and Se can act as free radical scavengers, affecting ROS elimination and also act as an antioxidant, improving the activities of oxidative enzymes resulting in increased antioxidative capacity in plants^51^. Many studies have shown that the protective role of Se and Si against oxidative stress in plants corresponded with the increase of oxidant enzyme activity and decrease of lipid peroxidation^22^. Increase in the activity of antioxidant enzymes such as CAT and APX as well as scavenging of H_2_O_2_ were also observed by this enzyme after Se and Si sprays^46^. Se increased plant growth, probably through increasing starch content in chloroplast, and due to its antioxidative properties, it protected cell membranes against lipid peroxidation^56^. Silicon also prevented access of proteases to internal membrane proteins and destruction and disturbance of cell membranes. Our findings further support those obtained by Jiang et al. and Tang et al. which reported that the application of exogenous Se and Si caused the expression of genes related to antioxidant defense and consequently increased the activities of SOD, CAT, and APX in maize and ramie plants ultimately increasing their drought tolerance^31,51^. These findings demonstrated that Se- and Si-mediated antioxidative defense enhancement was one of the important mechanisms protecting plants against oxidative stress due to drought.

### Growth, firmness and fruit yield parameters

Leaf number and leaf area in strawberry plants under drought stress were significantly decreased but spraying of nanoparticles reduced these negative effects. Decrease of leaf number and leaf area under drought stress has been reported in many species^57^. It is important to note these decreases result in reduced yield through a reduction in photosynthesis. Limitation of leaf area can be considered the first line of defense in dealing with drought; therefore, reduction of water potential during drought decreased water content of plant tissues which resulted in the decrease of leaf area^58^. The effect of water stress on cell development is more obvious because cell enlargement takes place after turgor pressure due to water absorption; therefore, any decrease in water content blocks growth^59^. Increase of cell number and area by Si^60^ and Se^61^ in some plant species has been reported. Hossain et al.^60^ reported that the increase in leaf size was not due to cell number increase but due to increased cell dimensions which indicated the effect of Si on cell elongation. It was found that Si and Se caused water loss from epidermis through deposition in epidermis cells of leaf and therefore preserved and maintained water in cells and increased turgor pressure resulting in the increase of green area of plants^62^.

The yield and yield parameters of strawberry plants decreased under drought stress although exogenous application of Se-, SiO_2_- and especially Se/SiO_2_-NPs modulated the negative effects of drought. Yield decreases in different species such as tomato^63^ and olive^2^ under drought stress has been reported. The decrease of yield under drought stress could be due to many reasons including decrease of photosynthesis efficiency, leaf area, assimilate production, and decrease of water and mineral absorption by the root which ultimately decrease developmental and vegetative growth^2^. Exogenous application of Si and Se modulated the unfavorable effects of environmental stresses on the yield of different species^4^. Research has shown that Se played a critical role in different plants, ultimately affecting plant yield, factors such as starch accumulation in chloroplast, resistance enhancement to oxidative stress, delaying of senescence, and water status adjustment under stress conditions and increase of antioxidative capacity^22^.

Drought stress decreased the firmness of strawberry fruits but spraying of nanoparticles increased this factor in plants under drought stress and normal conditions. This response could be due to the induction of ethylene production in fruits under drought stress^64^. Due to adjusting the expression of genes and enzymes involved in reactions related to cell walls, ethylene affects fruit firmness. Under the action of ethylene, the activity of polygalacturonase was increased and then decreasing fruit firmness. The positive effects of Si and Se on increasing fruit firmness has been reported for different species^65,66^. It seemed that the mechanical strength of plant tissues due to Si was generally because of amorphous solid Si deposition on cell wall layers^67^. On the other hand, the effect of Se on fruit firmness could be assigned to the prevention of different oxidative reactions in different fruits^68^.

### Fruit anthocyanin, total phenolic compounds, ascorbic acid content and antioxidant activity

The biochemical parameters of the fruit were severely affected by drought. Spraying Se-, SiO_2_- and especially Se/SiO_2_-NPs increased the contents of all three above mentioned biochemical parameters. Increase of anthocyanin and total phenolic contents under drought stress were observed in some plant species^69^. Anthocyanins are a group of phenolic compounds which comprise a large group of secondary metabolites and have antioxidant properties^70^. Polyphenol compounds are among antioxidant compounds which play their antioxidative roles through different mechanisms such as scavenging of free radicals, blocking oxidative reactions, hydrogen reduction, oxygen blocking reaction, metal ion chelating and acting as peroxidase enzyme substrate^71^. Increase of polyphenolic compounds under stress conditions is related to the genetic structure and growth environment of plants. Vitamin C is an organic acid; under drought stress, respiration is increased and therefore, these acids act as substrate in respiration phenomenon. This results in the decrease of acidity and consequently, decrease of vitamin C due to drought^72^. Increase in the amounts of biochemical parameters (anthocyanin, total phenolics, vitamin C and antioxidant activity) in fruits of different species by the addition of Si and Se has been reported^4^. Inorganic elements such as Si and Se play critical roles in determination of organoleptic properties and antioxidative capacity of fruits through adjusting the biosynthetic phenylpropanoid pathway which results in metabolite accumulation^73^. Antioxidants play protective roles against oxidative stress in plants, with free ^–^OH groups attached to the aromatic ring reducing oxidative damage by scavenging ROS and chelating metals. Generally, increases of secondary metabolites contributed to the improvement of cell responses to oxidative stress in addition to antioxidant activity of fruits which improved their quality^74^.

**Figure 5:**
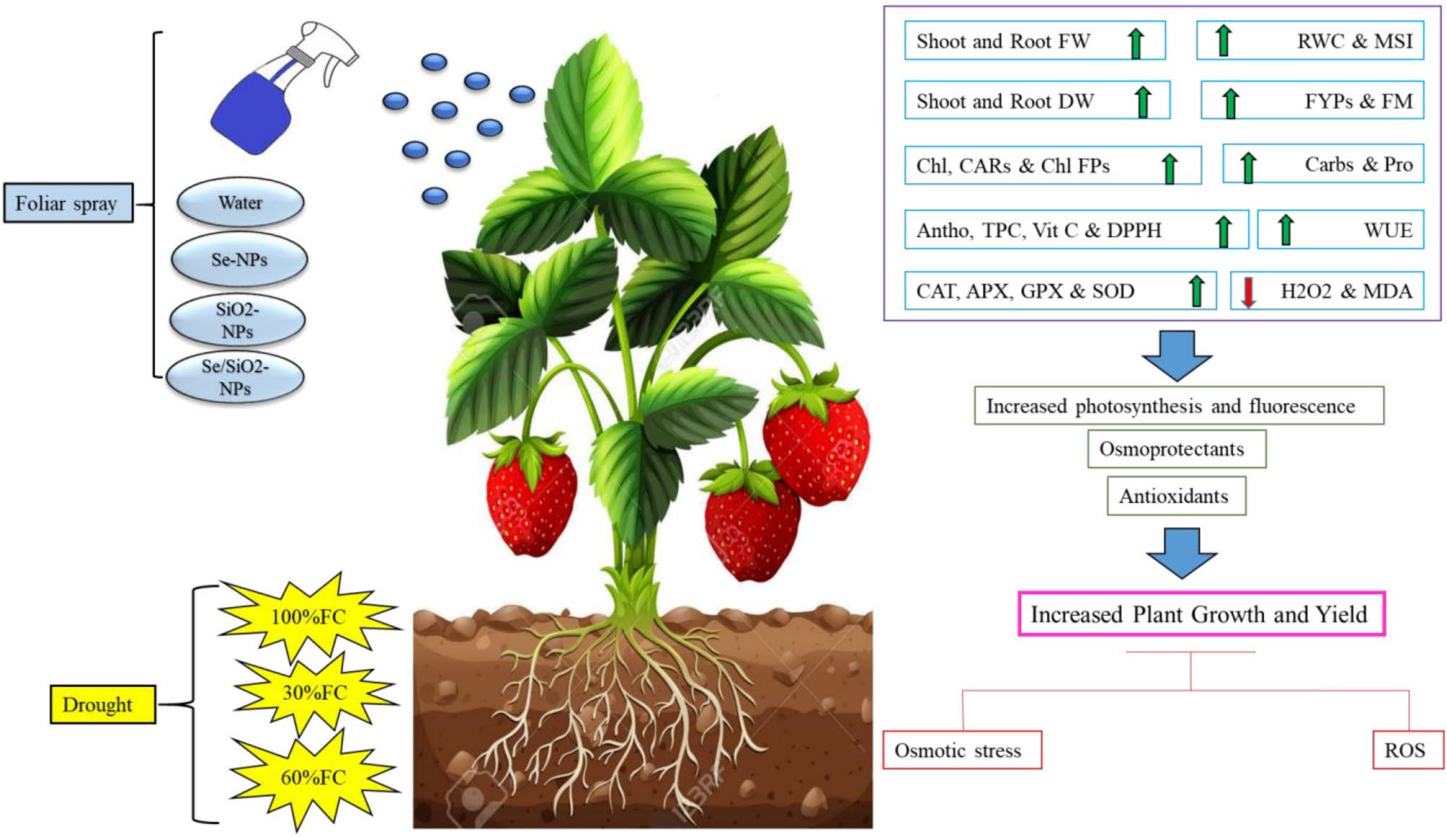
Schematic diagram illustrating the proposed mechanisms of selenium (Se), silicon dioxide (SiO_2_) and Se/SiO_2_ NPs-induced drought stress tolerance in strawberry plants. Application of Se-, SiO_2_- and Se/SiO_2_-NPs to drought stressed strawberry plants could improve growth performance and yield parameters by (i) protecting photosynthetic pigments and chlorophyll fluorescence to improve photosynthetic capacity, (ii) increasing proline and total carbohydrates for greater osmoprotection, (iii) activating antioxidant systems for maintenance of efficient reactive oxygen species (ROS) homeostasis, (iv) enhancing water use efficiency (WUE) level for improvement of root biomass and maintenance of proper osmotic status of the cells, and (v) accumulation of fruit biochemical compounds to increase fruit quality. *RWC* Relative water content, *MSI* Membrane stability index, *Chls* Chlorophylls, *CARs* Carotenoids, *Chl FPs* Chlorophyll fluorescence parameters, *Carbs* Total soluble carbohydrates, *Pro* Proline, *Antho* anthocyanin, *TPC* Total phenolic compounds, *Vit C* Ascorbic acid, *DPPH* antioxidant activity, *WUE* Water use efficiency, *CAT* Catalase, *APX* Ascorbate peroxidase, *GPX* Guaiacol peroxidase, *SOD* Superoxide dismutase, *H*_*2*_*O*_*2*_ Hydrogen peroxide, *MDA* Malondialdehyde.

In conclusion, spraying solutions containing Se-, SiO_2_- and especially Se/SiO_2_-NPs at 100 mg L^−1^ on strawberry plants is an efficient way to improve their drought tolerance and yield. Favorable effects of Se/SiO_2_-NPs on growth efficiency and yield parameters at different drought levels have been attributed to (1) protection of pigments to increase photosynthetic capacity, (2) accumulation of assimilates to protect osmosis, (3) activation of an antioxidative system to eliminate ROS, (4) enhancing water use efficiency level for improvement of root biomass and maintenance of proper osmotic status of the cells and (5) accumulation of fruit biochemical compounds (total phenolics, anthocyanin contents and antioxidant capacity) to increase fruit quality (Fig. 5). Since spraying of nanoparticles is easily performed, Se/SiO_2_-NPs sprays are advised for managing drought stress in strawberry and even other agricultural plants. However, many new studies are required for identification of the efficiency of Se/SiO_2_-NPs before practical programs can be realized on a large scale.

## Materials and methods

### Chemicals and reagents

**Figure 6:**
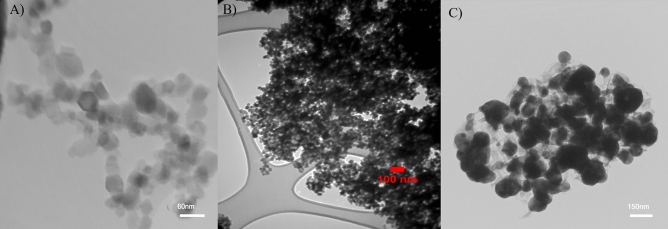
TEM images of (**A**) Se-, (**B**) SiO_2_- and (**C**) Se/SiO_2_ NPs.

**Figure 7:**
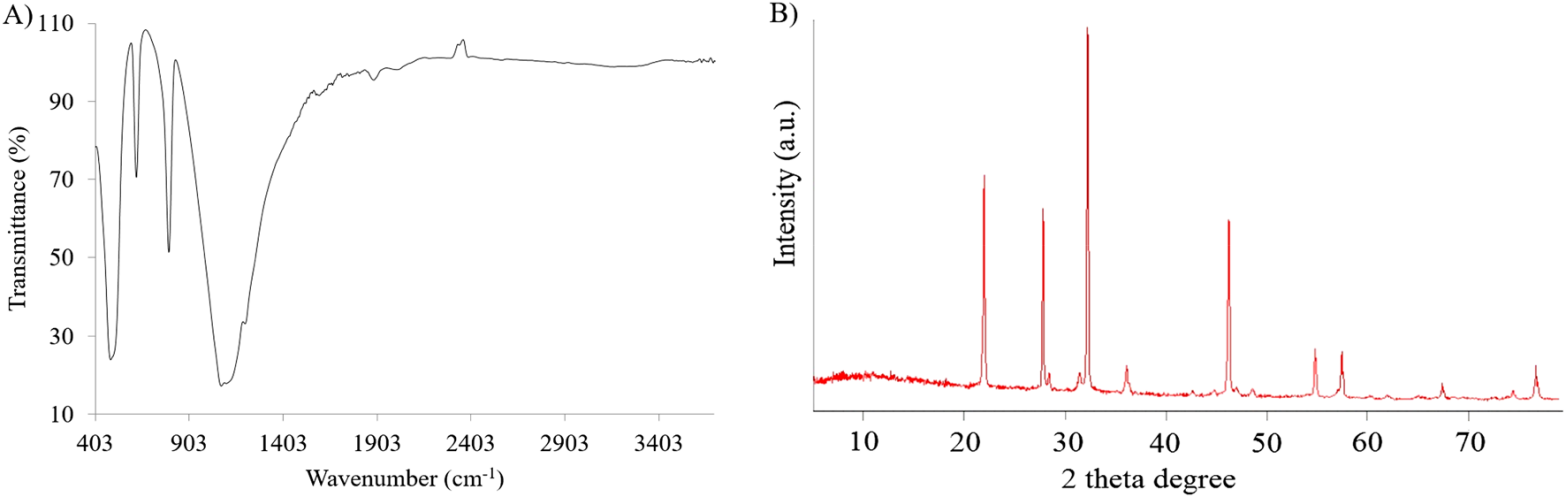
Characterization results of Se/SiO_2_ NPs applied in this work. (**A**) Fourier-transform infrared spectroscopy (FTIR), and (**B**) X-ray diffraction (XRD).

The silicon dioxide (SiO_2_-NPs) and selenium (Se-NPs) nanoparticle used in this study were obtained from the NANOSANY Corporation (Mashhad, Iran) (Fig. 6A,B). The Se/SiO_2_ nanoparticles were prepared at University of Maragheh Chemistry Lab (Supplementary File S2); characterization results of the Se/SiO_2_- NPs used in this study are presented in Figs. 6C and 7. The properties of SiO_2_-NPs, Se-NPs and Se/SiO_2_-NPs used in this experiment are shown in Supplementary File S3.

### Study site and treatments

The experiment was carried out from March 7, 2018 to June 21, 2018 in a greenhouse in Maragheh, Iran (37° 30′ N; 46° 12′ E, at an altitude of 1477.7 m; moderate to cold and relatively humid). The greenhouse photoperiod of 14/10 h (light/dark), temperature of 25/20 ± 5 °C (day/night) and air relative humidity of 80 ± 5% were maintained throughout the experiment. ‘Gaviota’ strawberry rooted plants (*Fragaria* × *ananassa* Duch.) were obtained from a strawberry nursery, Maragheh, Iran. These plants were planted in 7-L volume plastic pots, filled with about 525 g soil mixture (1:1:2 ratio of sand: animal manure: topsoil, respectively). Pots were irrigated by graduated cylinder daily to 100% field capacity (FC) for 15 days in the growing season (March 7th till March 22th). Then, three levels of watering were imposed that included normal irrigation (100% FC), moderate stress (60% FC), and severe stress (25% FC). The FC was determined by the gravimetric method following the methodology described by Souza et al.^75^. Preservation of the water treatments was made by daily weighing of the pots replacing the water lost by transpiration using a precision scale. These regimes were applied for 92 days in the growing season (March 22th till June 21th) until harvest. Plants (5 fully expanded leaves) were subjected to non-drought (control) and different levels of drought (moderate and severe). At day 35 after the start of the drought treatment plants did show stress symptoms. On that day, the upper leaf surfaces of control and drought-treated strawberry plants were sprayed until full wetting (ca. 25 mL plant^−1^) with solutions containing 0 (NPs-untreated control), Se-NPs (25 mg L^−1^), SiO_2_-NPs (125 mg L^−1^) and Se/SiO_2_-NPs (50 and 100 mg L^−1^) once a week until harvest (3 sprays in total). Foliar application was done before the flowering stage and at sunset. Before each exposure, solutions were prepared with NPs dispersed in ultrapure water and homogenized 20 min with an ultrasonic bath. Plants from each treatment were harvested for evaluating their morpho-physiological responses.

### Leaf photosynthetic pigments and chlorophyll fluorescence

Chlorophyll a (Chl a), Chl b, total Chl and carotenoids (CARs) were measured in leaf samples according to the method of Arnon (1949) using a spectrophotometer (Shimadzu, Model UV 1800, Kyoto, Japan) at 470, 663 and 645 nm, respectively^76^.

Chlorophyll fluorescence parameters were assessed using a portable photosynthesis meter (Walz GmbH Eichenring, 691090 Effeltrich, Germany) at the end of treatments. Minimal fluorescence, *F*_*0*_, was measured in 30 min dark-adapted leaves and maximal fluorescence, *F*_*m*_, in the same leaves in full light-adapted conditions. Maximal variable fluorescence (*F*_*v*_) and the photochemical efficiency of PSII (*F*_*v*_*/F*_*m*_) for dark adapted leaves were also calculated from the measured parameters^77^.

### Leaf total soluble carbohydrates, relative water content, membrane stability index and proline

To measure soluble carbohydrates, 100 mg FW (fresh weight; FW) of leaf tissue was homogenized with 10 mL of 95% ethanol, and extracts were centrifuged at 6000×*g* for 15 min. Then, the upper phase of the centrifuged samples was supplemented with 3 mL of anthrone and maintained at 100 °C for 10 min in boiling water. Then, the absorbance at 630 nm was read using a spectrophotometer (Shimadzu, Model UV 1800, Kyoto, Japan)^78^. Total soluble carbohydrates were determined using a glucose standard, and expressed as mg g^−1^ FW. Relative water content (RWC) was measured as described by Barrs and Weatherley^79^. The membrane stability index (MSI) was determined according to methods established by Sairam et al.^80^. Proline was measured spectrophotometrically by the method of Bates et al.^81^.

### Leaf malondialdehyde contents and hydrogen peroxide

Malondialdehyde (MDA) content was measured in leaf samples spectrophotometrically at 532 nm according to Dhindsa et al.^82^. The reaction of hydrogen peroxide (H_2_O_2_) to potassium iodate was assessed spectrophotometrically at 390 nm according to Alexieva et al. to quantify the H_2_O_2_ content in leaf samples with the help of an H_2_O_2_ standard curve^83^.

### Leaf catalase, ascorbate peroxidase, guaiacol peroxidase and superoxide dismutase

To determine catalase (CAT; EC 1.11.1.6) activity in leaf extracts, 30 µl of extract were added to 50 mM K phosphate buffer (pH 7.0) and 2% H_2_O_2_ for a total volume of 3 mL. Enzyme activity was recorded at 240 nm for 2 min with a spectrophotometer (Shimadzu, Model UV 1800, Kyoto, Japan)^84^. Ascorbate peroxidase (APX; EC 1.11.1.11) activity was assayed according to the methods of Nakano and Asada^85^. 1.95 mL reaction mixture contained in 50 mM K-phosphate buffer (pH 6.0), 0.5 mM ascorbate, 0.1 mM EDTA-Na_2_, 1.0 mM H_2_O_2_ and 65 μL of enzyme extract. The increase in absorbance was recorded at 290 nm after 4 min. The activity of guaiacol peroxidase (GPX; EC 1.11.1.7) was assayed following the method of Chance and Maehly^86^. The reaction mixture contained 50 mmol L^–1^ phosphate buffer (pH 7.0), 0.1 mmol L^–1^ guaiacol, 0.1 mmol L^–1^ H_2_O_2_ and the enzyme aliquot. Enzyme activity was measured by the increase in absorbance at 470 nm caused by guaiacol oxidation [E = 26.6 mmol (L cm)^–1^]. Superoxide dismutase (SOD; EC 1.15.1.1) activity was measured by estimating its ability to prevent the photochemical reduction of nitro-blue-tetrazolium (NBT) according to Beauchamp and Fridovich^87^. The SOD activity was measured spectrophotometrically at 560 nm.

### Growth, firmness and fruit yield parameters

The plant growth parameters were investigated at the end of experiment by recording the number of leaves and leaf area (using Image J Software). Also, the number of inflorescences, flowers and fruits as well as fruit size was recorded. Fruit yield was determined in grams per plant. Fruit weight was also measured by digital balance with accuracy of 0.1 g.

Firmness was determined on the equatorial region of fruit, using texture analyzer (STEP Systems GmbH, Nuremberg, Germany; with an 8 mm probe).

### Fruit anthocyanin, total phenolic compounds, ascorbic acid content and antioxidant activity (DPPH)

Tissue samples were obtained from various fruit parts and frozen in liquid nitrogen. Then, 5 g tissue was homogenized in 10 mL 50 mmol L^–1^ phosphate buffer at pH 7.8. The homogenate was centrifuged at 15,000×*g* at 4 °C for 20 min and the supernatant (fruit extract) was collected to measure fruit quality parameters:

Total anthocyanins were estimated by the pH differential method using two buffer systems: 25 mM KCl buffer (pH 1.0) and 0.4 M Na acetate buffer (pH 4.5). Samples were diluted with KCl buffer until A510 was within a linear range of the spectrophotometer. The same dilution factor was later used to dilute the sample with Na acetate buffer. Readings were performed after incubating for 15 min at 510 and 700 nm in the two buffers, five replications per sample, and total anthocyanin content was calculated as indicated by Giusti and Wrolstad^88^:

$$ \left[ {\left( {{\text{A }} \times {\text{ MW }} \times {\text{ DF }} \times { 1}00} \right)/{\text{MA}}} \right] $$

where A = (A510 – A700); MW = molecular weight; DF = dilution factor; MA = molar absorptive coefficient of cyanidin-3-glucoside (C_3_G). Results were expressed as mg C_3_G 100 g^–1^ of juice.

For phenolic compound analysis, 100 μL of fruit extract was mixed with 400 μL phosphate buffer and 2.5 mL of Folin reagent (Sigma‐Aldrich). After 1 min, 2 mL of Na_2_CO_3_ (7.5%) was added to the mixture and the sample kept at 50 °C for 5 min, before measuring the absorbance at 760 nm with a spectrophotometer (Shimadzu, Model UV 1800, Kyoto, Japan). Gallic acid was used as a standard, and results were expressed as mg of gallic acid per 100 g FW^89^.

The ascorbic acid (Vit C) concentration in fruit extracts was determined by titration using a solution containing I and KI (16 g KI and 1.72 g I in 1 L water). The titration ended when the sample turned dark blue and color was stable. The volume of the I + KI solution was recorded and the concentration of ascorbic acid calculated according to the following equation as ([0.88 × V]/5 × 100), where V is the volume of the consumed I + KI solution^90^.

The DPPH (2,2-diphenyl-1-picrylhydrazyl) free radical scavenging activity was determined according to the method reported by Brand-Williams et al.^91^. The absorbance was read at 517 nm with a spectrophotometer.

### Water use efficiency

Water use efficiency (WUE) was calculated as marketable fruit yield per unit water used^92^.

### Statistical analysis

Analysis of variance (ANOVA) was applied on the obtained data through GLM procedure of Statistical Analysis Software (SAS, version 9.1). Duncan Multiple Range test was performed to obtain significant differences among treatments at the significance level of *P* < 0.05. Pearson correlation coefficient, dendrogram clustering and principal component analysis (PCA) were performed using R v3.4.3 (www.r-project.org).

## Supplementary information

Supplementary Information.
