# Supplementary material for: Selenium and silica nanostructure-based recovery of strawberry plants subjected to drought stress
Source: Sci Rep. 2020 Oct 19;10:17672. doi: 10.1038/s41598-020-74273-9 (PMC7572471; doi:10.1038/s41598-020-74273-9)
Supplement: Supplementary file 1 — Supplementary Information. [file 41598_2020_74273_MOESM1_ESM.doc]

**Selenium and silica nanostructure-based recovery of strawberry plants subjected to drought stress**

Seyed Morteza Zahedi^a^,*, Faezeh Moharrami^a^, Saadat Sarikhani^b^, Mohsen Padervand^c^

^a^ Department of Horticultural Science, Faculty of Agriculture, University of Maragheh, Maragheh, Iran. E-mail: [s.m.zahedi@maragheh.ac.ir](mailto:S.M.Zahedi@maragheh.ac.ir)

^b^ Department of Horticulture, College of Aburaihan, University of Tehran, Tehran, Iran

^c^ Department of Chemistry, Faculty of Science, University of Maragheh, Maragheh, Iran

***Correspondence**:

[s.m.zahedi@maragheh.ac.ir](mailto:S.M.Zahedi@maragheh.ac.ir) (S.M. Zahedi); Fax: +98 41 37278001; Tel: +98 914 3528813


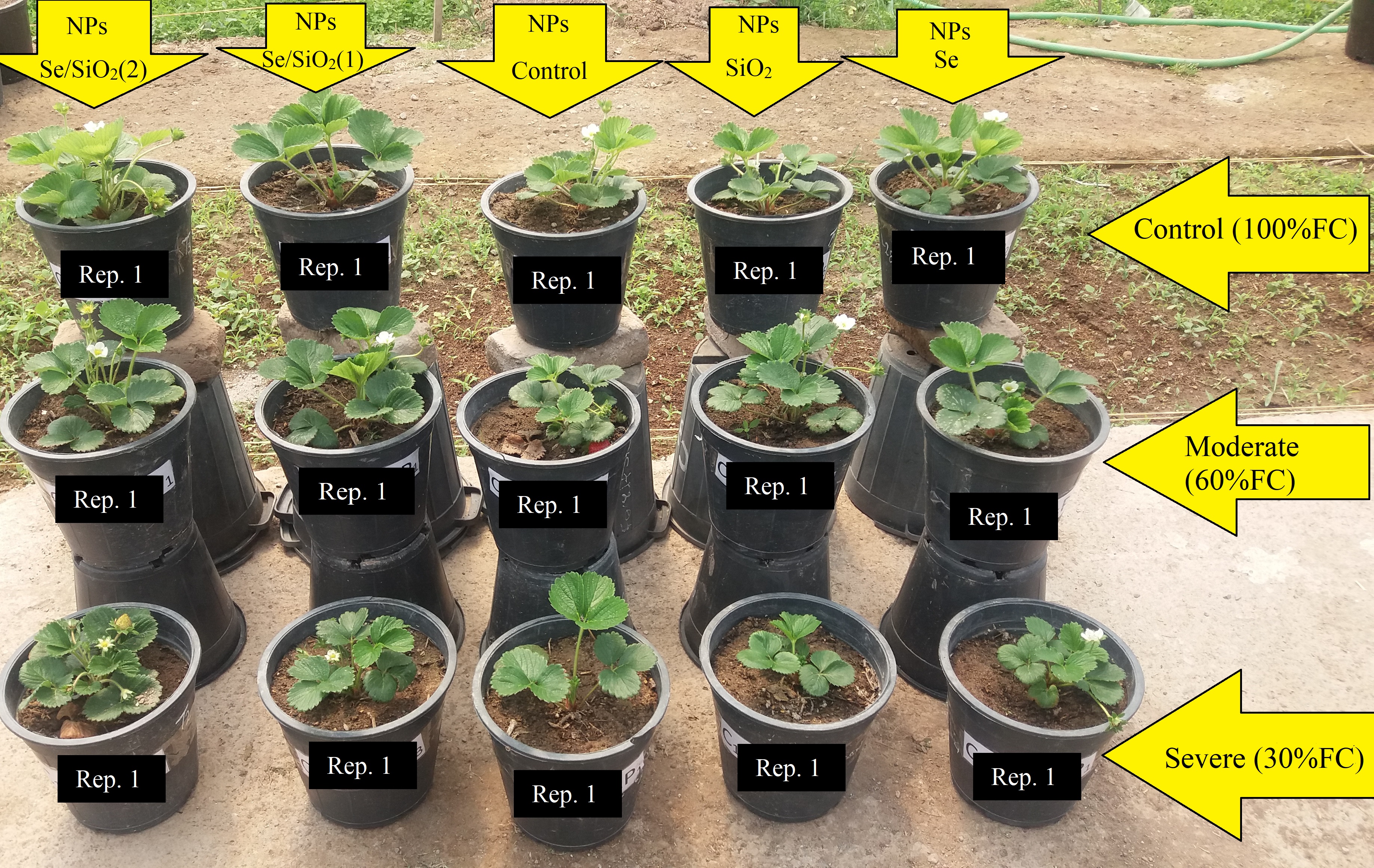


**S1)**

Strawberry plants under drought stress and selenium nanoparticles (Se-NPs), silicon dioxide nanoparticles (SiO_2_-NPs) and Se/SiO_2_-NPs foliar spray

**S2**)

To prepare Se/SiO_2_-NPs, we acted as follow:

EP powder (2.5 g) was dispersed in 3M NaOH solution. The mixture was then refluxed in a 250 mL 3-neck flask equipped with a reflux condenser at 100 °C under magnetic stirring for 24 h to obtain sodium silicate. The as prepared slurry was filtered and washed with minimum volume of boiled distilled water. 5 M HCl was added to the mixture under magnetic stirring until the medium became neutral. The soft and white aqua-gel product was kept at room temperature for 24 h followed by a drying period of 24 h at 80 °C in an oven. The obtained white precipitate was calcined at 800 °C for 4 h under air ventilation. The product is SiO_2_-NPs.

For Synthesis of Se loaded ionic liquid ([MOYI]Cl)-grafted SiO_2_ support, 1 g of the as-prepared powder was dispersed in 30 mL of distilled water containing 0.1 mL of [MOYI]Cl and 0.05 g glucose (as reducing agent). After 45 min of stirring, an aqueous solution of Na_2_SeO_3_ (15 mL, 0.05 M) was gradually added at 50 °C and the mixture was stirred for a further 60 min. After completion the reaction, the precipitate was filtered, washed with distilled water three times, and then dried at 60 ºC overnight. The final product is Se/SiO_2_-NPs.

**S3**)

Some properties of nanoparticles (NPs) with the structure of selenium and silica in this experiment

|  | **Se-NPs** | **SiO_2_-NPs** | **Se/SiO_2_-NPs** |
| --- | --- | --- | --- |
| Purity | 99.95% | 99+% | Synthesized sample, not defined |
| APS | 10 - 45 nm | 20-30 nm | SiO_2_: 50-80 nm, Se: 5-20 nm |
| SSA | 30 - 50 m^2^/g | 180-600m^2^/g | 24.5 m^2^/g |
| Color | Crimson | White | White |
| Morphology | Almost spherical | Amorphous | Crystalline |
| True density | 3.89 g/cm^3^ | 2.4 g/cm^3^ | Synthesized sample, not defined |

Average particle size, APS; Specific surface area, SSA; Selenium nanoparticles, Se-NPs; Silicon dioxide nanoparticles, SiO_2_-NPs
